# Patient Perspectives in Geographic Atrophy (GA): Exploratory Qualitative Research to Understand the Impact of GA for Patients and Their Families

**DOI:** 10.22599/bioj.137

**Published:** 2019-11-12

**Authors:** Jill Carlton, Sarah Barnes, Annette Haywood

**Affiliations:** 1 University of Sheffield, GB

**Keywords:** Geographic atrophy, quality of life, qualitative, focus group, age-related macular degeneration

## Abstract

**Background:**

Age-related macular degeneration (AMD) is the major cause of blindness for the older population in the developed world. Geographic atrophy (GA) is an advanced form of AMD. This progressive degenerative disease causes loss of visual function but unlike exudative AMD there are currently no approved therapeutic treatments for GA. Instead management of the condition is through supportive care. The aim of this study was to conduct exploratory qualitative research to develop a further understanding specifically of the impact of geographic atrophy (GA) on the quality of life for both patients and their families and to explore the resources GA patients most frequently access.

**Methods:**

Two focus groups were conducted where participants were assigned to the ‘better’ or ‘worse’ group based upon their level of visual acuity. The data were analysed using the principles of thematic analysis. Transcripts were coded using an a priori framework. Emerging themes and subthemes were added, and transcripts recoded to reflect this. Transcripts were coded by one researcher, and the emerging themes and subthemes discussed and agreed prior to transcript recoding.

**Results:**

Nine participants were recruited to the study (n = 5 ‘better group’ and n = 4 ‘worse group’). Six overarching themes were identified. These are: experience of symptoms and understanding of GA; impact on activities; feelings and emotions; relationships and informal support; accessing formal support; and information needs.

**Conclusion:**

Key findings indicated that: participants had little knowledge of the mechanisms of GA but were aware of their prognosis; activities impacted by GA included management of daily activities and reading; emotions experienced included fear and frustration, and these frequently crossed over into their relationships with others; and access to formal support was mainly practical and information needs were largely unmet, with peer support being identified as important. Management of GA should include signposting to appropriate support agencies, such as low-vision services and charities.

## Background

Age-related macular degeneration (AMD) is estimated to affect over 50 million individuals worldwide (Barnett & Handa 2013), and is the most common cause of blindness in developed countries (Bourne et al. 2014). Geographic Atrophy (GA) is an advanced form of AMD, affecting over 5 million people worldwide (Wong et al. 2014), and in the United Kingdom (UK) is estimated to account for 26% of legal blindness (Rees et al. 2014). The incidence of GA increases exponentially with age (Rudnicka et al. 2012; Wong et al. 2014). This progressive degenerative disease causes loss of visual function but, unlike exudative AMD, there are currently no approved therapeutic treatments for GA. Management of the condition is through supportive care, i.e. referral to low vision clinics for assessment, advice, and supply of equipment and, in some situations, notification to social services.

GA lesions usually initially develop near the fovea, without involvement of the foveal centre, but progress over time until the fovea is involved (Sunness 1999). Best corrected visual acuity (BCVA) is one way in which studies have described the impact of GA on an individual. However, BCVA may not be a good reflection on the impact GA has on the individual, as some people can have good levels of vision before the fovea is involved, while still experiencing problems related to other aspects of visual function (such as reading speed, low contrast sensitivity, reduced night vision) (Sadda et al. 2016). The experience of AMD is well-reported (Burton et al. 2015; McCloud et al. 2014; Taylor et al. 2016). In a systematic review and meta-analysis of qualitative research Bennion et al identified six themes relating to emotional impact of AMD; functional limitations, adaptation and independence; the future with vision impairment; social engagement; disclosure; and interaction with health services (Bennion et al. 2012). However, the authors acknowledged that caution in interpreting the results must be given, as some of the studies reported contradictory findings particularly with respect to feelings and emotions. Some of the studies included in the review suggested positive emotions such as hope and optimism, which contrasted with other studies where participants raised negative emotional comments about their experiences Furthermore, little (if any) information detailing the cultural and societal support of the individuals within each of the included studies was provided. Of the eight studies included in their review, only one was conducted in the UK, and none provided information on ethnicity of the study participants. The other studies included in the review were conducted in Australia (n = 1), Sweden (n = 1) and the United States (n = 5). The review and other studies that report the impacts of AMD on an individual often include participants with exudative (wet) AMD, or do not distinguish between those with exudative or GA. The influence of this on findings may be significant, since the clinical course of exudative AMD and GA are quite different (Stanford et al. 2009). The aim of this study was to develop and conduct exploratory qualitative research to develop a further understanding specifically of the impact of GA on quality of life (QoL) for both patients and their families; and to explore the resources GA patients most frequently access, as they currently have no effective treatment options.

## Methods

The research had all appropriate NHS ethical and governance approval and was reviewed by NRES Committee North-East (Ref: IRAS 227791). Data collection was carried out in October 2017.

### Sampling and recruitment

Participants were recruited via Sheffield Teaching Hospitals NHS Foundation Trust (STH), UK, through two sources: new patients attending clinic appointments and an existing database of patients with GA. Potential participants attending clinic appointments who satisfied the inclusion/exclusion criteria were given an information sheet and consent form. Potential participants identified via the database were sent an invitation letter, information sheet, and consent form.

Members of the clinic team at STH identified individuals matching the following sampling framework in an attempt to ensure a good variation within potential participants in terms of age, duration of GA and gender. Information was recorded on whether unilateral or bilateral GA was present; how long the participant had been diagnosed with GA; and participant’s VA.

Inclusion criteria:

- unilateral or bilateral GA diagnosed by an ophthalmologist
- able to read and speak fluent English (without the need for an interpreter)

Exclusion criteria:

- presence of choroidal neovascular membrane (CNV)
- advanced diabetic eye disease
- significant loss of visual field or VA not related to GA (e.g., from glaucoma, stroke, diabetic eye disease, etc.)
- current or have previous intraocular treatment for GA through a clinical trial

All potential participants were contacted by the STH team approximately 1–2 weeks after receiving the information. The purpose of the phone call was to allow potential participants the opportunity to ask any questions about the study, and for the STH team to determine whether the individual was making an informed decision about whether they wished to participate or not. At the end of the phone call, the STH team ascertained that the potential participant was capable of completing the consent form, and if they were willing to participate, asked that it be returned to STH in the stamped, addressed envelope. Once the completed consent form was received, the STH team accessed the individual’s hospital records and completed a Participant Condition Form. This provided information on whether the individual should be allocated to the ‘better’ (visual acuity (VA) of 6/6 to 6/18) or ‘worse’ vision (VA worse than 6/18) focus group in an attempt to mitigate potential distress to participants. A letter was then sent to the individual confirming the date, time and venue of the focus group.

Before the focus group began, participants were asked to complete a Demographic Information Form, which included any self-reported comorbidities. If they were unable to complete either of these forms independently, help was provided by the research team. Informed consent was obtained again at this time. The focus groups were facilitated by two experienced qualitative researchers (SB and AH).

## Results

### Sample

Nine participants took part in the study, and the majority of participants were female (n = 8). Details of participants’ clinical characteristics are given in Table 1. Further details of the sample are reported in Tables 2 and 3.

**Table 1:** Participant clinical characteristics.

Participant	Age (years)	Date of diagnosis of GA	Time since diagnosis (months)	Type of GA	Date of last recorded BCVA	Visual Acuity		Details of any other ocular condition	OCT details	
	
						RE	LE		Focal/Multifocal	Involve fovea?

Female 1	72	6/10/17 (BE)	1	Bilateral	6/10/17	6/60	6/36	Nil	Focal (BE)	Yes (BE)
Female 2	87	29/12/14 (RE) 29/9/17 (LE)	34	Bilateral	29/9/17	3/60	6/36	Nil	Focal	Yes
Female 3	71	5/8/16	14	Bilateral	21/8/17	6/36	6/36	Nil	Focal	Yes
Female 4	82	15/5/15	29	Bilateral	14/9/17	1/60	6/24	Nil	Multifocal	Yes
Female 5	88	28/4/17	6	Bilateral	7/8/17	6/9	6/18	Bilateral pseudophakia	Focal (LE) Multifocal (RE)	Yes
Female 6	87	7/9/17	1	Unilateral	7/9/17	6/12	6/18	Bilateral pseudophakia	Multifocal	Yes
Female 7	79	17/3/17	7	Bilateral	11/8/17	6/12	6/60	Nil	Focal (LE) Multifocal (RE)	Yes (LE)
Female 8	81	11/8/17	2	Bilateral	25/9/17	6/12	6/24	Nil	Focal	No
Male 1	79	23/10/15	24	Bilateral	26/5/17	6/9	1/60	Bilateral pseudophakia	Focal	Yes

BCVA – best corrected visual acuity; RE – right eye; LE – left eye; OCT – optical coherence tomography.

**Table 2:** Demographic information and self-reported comorbidities: ‘worse group’.

Participant	Age (years)	Living arrangements	Registered blind/partially-sighted/no registration	Any other condition(s)

Female 1	72	With partner	Partially sighted	High blood pressure
Female 2	87	With partner	Partially sighted	Stroke, arthritis
Female 3	71	With partner	Partially sighted	Asthma, thyroid problems, osteoporosis
Female 4	82	With partner	No registration	Chest or lung disease, arthritis, IBS, interstitial cystitis

**Table 3:** Demographic information and self-reported comorbidities: ‘better group’.

Participant	Age (years)	Living arrangements	Registered blind/partially-sighted/no registration	Any other conditions(s)

Female 5	88	With partner	No registration	COPD, arthritis
Female 6	87	Alone	No registration	Blood pressure, under active thyroid
Female 7	79	With family	No registration	Asthma
Female 8	81	Alone	No registration	Arthritis
Male 1	79	With partner	Not answered	Cancer

Each focus group lasted approximately 90 minutes. The discussion was informed by a topic guide (see Supplementary Material), derived using evidence from the literature and clinical input from the funder. Participants were given a £40 shopping voucher to reimburse their time and travel expenses. The focus groups were audio recorded and transcribed verbatim. The data were analysed using the principles of thematic analysis, with the assistance of the computer software programme NVivo10. Transcripts were coded using an a priori framework based on the topic guide. Emerging themes and subthemes were added, and transcripts recoded to reflect this. Transcripts were coded by one researcher, and the emerging themes and subthemes discussed and agreed with a second researcher prior to transcript recoding.

Six overarching themes were identified. These are: experience of symptoms and understanding of GA; impact on activities; feelings and emotions; relationships and informal support; accessing formal support; and information needs.

### Experience of symptoms and understanding of GA

Participants in both groups were affected by a range of symptoms from GA with most experiencing varying levels of disrupted peripheral and central vision; intolerance to brightness and glare; dizziness and problems with face recognition.

‘Like when I’m getting tablets out, I can look and think, I’ve only got one and then I’ll look again and I’ve got two … It’s difficult to differentiate things.’ (Female 3; worse)

‘It’s just looking straight forward at somebody; all your faces look … mingled.’ (Female 8; better)

All the participants knew where they could find practical support and advice and had access to glasses or aids supplied by opticians and magnifying glasses or other aids supplied by low vision clinics.

‘… that lady that comes out to see you … they’ve got these orange stickers and they stick you them on your cooker and things like that.’ (Female 2; worse)

Participants had little understanding of the mechanisms of GA with some suggesting it started as a result of a cataract operation, and others assuming it was part of the natural ageing process. Some participants were unaware of why they had been referred to the eye clinic in the first instance. However, the participants did appear to be more knowledgeable about their prognosis and were aware that, while there was no known cure for GA, they were unlikely to go completely blind.

‘I thought it was just age. Because I’ve always been a voracious reader and I thought oh it’s age.’ (Female 5; better)

‘All I know is that … or what they told me was that you lose central vision but keep peripheral … that’s as much as I know.’ (Female 6; better)

### Impact on activities

The negative impact of symptoms of GA on activities largely fell into two main areas: management of activities of daily living; and reading (both for pleasure and for need). Participants described coping strategies they had learnt in order to maintain independence or continue with their activities and hobbies. Symptoms such as blurring, distortion and changes in perception affected activities such as driving, travelling as a passenger in a car and navigating on foot. Several participants had given up driving, either voluntarily or because of a directive from the eye clinic. Symptoms were also experienced when travelling as a passenger in a car driven by someone else.

‘And when I’m travelling with my husband he will insist on saying, did you see what it said on that sign? And I’ll say, no because I can’t focus when I’m moving.’ (Female 1; worse)

Walking was affected by distorted perception, and using public transport was described as problematic, both in terms of reading bus numbers from a distance and knowing when to get off the bus.

‘I was out with my daughter once and you know your perception of things, I was in the winter garden with her and erm I said you can’t go out there there’s a yellow band in the way stopping us going she said Mum that’s on the floor it’s to walk over.’ (Female 3; worse)

‘Crossing the road. I can’t see cars coming from a distance. I can see when they’re near, but that’s too late then if you’ve crossed over so I have to wait and I’ve brought my stick with me today cos I knew I’d got to cross some roads ….’ (Female 8; better)

‘But I got on the bus … today and I said to the driver … I can’t read because I’ve got erm, a macula problem, I says and I can’t see and he says go and sit down love … It stopped and he says here you are love, you want to be off here, you know and, and that were lovely.’ (Female 4; worse)

All participants had issues with reading since the onset of GA. Many participants had reluctantly given up reading for pleasure, mainly because it took a lot longer.

‘I used to love *Star* newspaper, I loved that paper it’s all local but I can’t read it.’ (Female 2; worse)

‘I just miss being able to read my books … I used to love the feel and the smell of, cuddling down in bed with a book … I mean going into the library and just selecting a book and erm … I’ve read all my life you know, and I, I just miss it so much.’ (Female 3; worse)

Despite having access to aids to support reading the problems experienced were mainly related to the time it took to follow the text and make sense of it.

‘Like I say I’ve got one of those magnifying glasses but by the time I’d read it it’d take me ten minutes … and then your eyes are aching.’ (Male 1; better)

‘Well I used to read the, the lesson at church and erm … I had to start using me [magnifying] glass, but, I couldn’t read the words fast enough, through the glass, to, to move the glass along … I couldn’t read to make the words make sense. I’ve stopped midway between the sentence and then I’d start again and I’m thinking it don’t make sense that bit, you know.’ (Female 4; worse)

When it came to reading for necessity, participants had difficulties with reading menus, reading sell-by dates, prices and other information on products, and using card machines in shops.

‘I mean if I go out to a restaurant or anything, you can’t, you can’t see menu and I keep saying what have they got on and it doesn’t seem the same when they’re telling you, as what it does when ….’ (Female 2; worse)

‘You can’t see dates on … on you know when you’re picking parcels, whatever you’re picking up … you can’t the dates on them at all. Is it out of date? Isn’t it? You can’t see that, that sort of thing.’ (Male 1; better)

‘But that’s the way I am but some people can’t, I’ll tell you what I do find in shops, you know, if you’ve got your card and you shove it in, sometimes you just can’t see the numbers to press in.’ (Female 3; worse)

Other activities that participants had given up, or had difficulty with, because of the symptoms of GA were housework, sewing and craft, puzzles, reading music and watching TV. Most of the participants had adopted strategies in their determination to help them maintain their activities and hobbies. These strategies involved using aids/gadgets, adapting tasks and relying on other people for help.

‘I’ve had to get a Kindle you know and make the writing bigger.’ (Female 3; worse)

‘I get the hook and I stick it in that finger there so I know where the point is and then I get either me pellet or me maggot and I can’t see it I have to feel it … I’m devising different things so I can carry on going [fishing]….’ (Male 1; better)

### Feelings and emotions

Participants experienced a range of emotions since the onset of GA. Anger and particularly frustration were emotions participants related to GA, often when trying to carry out tasks which had previously been straightforward. These emotions were also often conveyed onto their relationships with others.

‘Same when I’m fishing I get so mad with myself at times I should be able to do that and this and I should be able to see that and I can’t and when you’ve always had good eyesight shall I say and suddenly this is coming on it gets you so … so frustrated.’ (Male 1; better)

‘Oh. I get frustrated when I can’t do things what I used to do.’ (Female 1; worse)

Participants spoke of feeling frustrated about their lack of independence, coupled with the frustration of having to seek assistance from others. Fear, particularly in relation to deterioration of their eyesight, was another emotion frequently expressed by participants.

‘My only concern is I’d like it to not get any worse than this … if you know what I mean. If it did get worse well you know I think it’d be frightening if you were going to really go … If it stopped like this I could cope with it ….’ (Male 1; better)

‘I think my greatest worry is wondering exactly how bad it will get, as to how well I’ll be able to manage, but most of the time I just ignore it at the moment.’ (Female 6; better)

As their condition deteriorated, many of the participants worried about losing independence and becoming reliant on others. In one participant, this worry of managing alone was exacerbated because of a recent bereavement.

‘… the only thing I’m worried about is being left on me own … Because I haven’t got somebody nearby enough to be with me all the, you know to come every day … Because you get to the stage where you can’t do things and you can’t balance ….’ (Female 2; worse)

‘What upset me was erm I had to give up driving. I’m such an independent person I have been all my life and I hate having to ask people.’ (Female 4; worse)

‘I’m coming to terms with it a little because my husband died this year … he brought me everywhere and I never used to realise how bad my eyes were until he’d gone … That’s when it hit me.’ (Female 8; better)

### Relationships and informal support

The participants were getting informal support from a variety of sources including their spouse and/or grown up children.

‘Yes, yes he does, he’s quite helpful, he’s quite good at cooking … He makes some messes but, you know overlook that don’t you.’ (Female 1; worse)

‘I mean my kids are wonderful, they’re there if I need them.’ (Female 3; worse)

‘My husband’s wonderful … But … he likes to be in charge … he insisted on doing all my tablets you know, until it got to a point said, I know what I’m doing now and I had to probably take control back off him, but he really does want to help …’ (Female 4; worse)

For those participants who didn’t have family members close by, friends and neighbours provided invaluable support. The participants were generally appreciative of the support provided to them by their friends and family. However, this appreciation was in some cases accompanied by frustration at a loss of independence and a need to take back control from others.

‘…you know he’s marvellous but it does get on your nerves … they take your life over it’s awful to say it but I wish, he’d go out for the day and leave me alone ….’ (Female 2; worse)

‘… she’ll say now mum … have you thought about this and don’t forget you’ve got to do that. And I say yes darling I know … and sometimes I get a bit frustrated ….’ (Female 7; better)

It was apparent from the participants that, as their condition deteriorated, family members began to realise the potential consequences and roles and responsibilities began to change. This could manifest, for example, after bereavement when adult children were drawn on for further support. The impact of the condition on informal carers was particularly evident on those who had chronic conditions themselves and participants were aware of the implications of this.

‘My daughter’s already planning this. They just bought a new house and they’re planning for when … if I can’t see if it gets worse I’ve got me own place in their house ….’ (Female 8; better)

‘We sat round the breakfast table in the dining room and I couldn’t see where the salt and pepper was and she were having to show me… Yeah and I think then it dawned on them. … then they realised it were worse than what they thought.’ (Female 8; better)

‘… he’s had prostate cancer and so he has a stoma and he gets a bit agitated at times … I mean he’s, he’s taking us three places this week … each day I’ve been out and he’s had to take me and, he does say I’m sick of waiting about you know.’ (Female 4; worse)

### Accessing formal support

The participants had access to a range of health professionals and allied health professionals. They were generally satisfied with their experience at the hospital eye clinic and low-vision clinic, and had particular confidence in the specialist nurse.

‘What I do like about the eye clinic up at erm the [hospital] is that they always send the letters out on yellow paper … It’s absolutely marvellous.’ (Female 4; worse)

‘And … there is the emergency clinic if you suddenly think ooh something’s happened with my eyes, you can, you can go.’ (Female 3; worse)

‘… she said if you get any change whatsoever you come straight back and I thought what is she expecting? I was really frightened and erm and then when … we made another appointment so I went after I came back from holiday and she said … I’m discharging you but any change whatsoever you come straight back to A&E.’ (Female 7; better)

Some participants noted that they were originally uncertain of why they had been referred to the eye clinic in the first instance.

‘It were Specsavers who er referred me back although I’d been discharged from them up at the [hospital] after having the cataracts done er and she was doing me glasses and she says I’m going to refer you back to the hospital.’ (Female 6; better)

‘It was my GP … She’s just left and she was an angel … and I says I’ve had this deterioration she says go to the walk-in she’d know then.’ (Female 5; better)

A couple of the participants had been accessing support from charitable organisations in the city. These organisations offered social support as well as providing some gadgets and aids for low vision conditions.

‘I go to the SRBS^1^ … and they’ve been fantastic they’re so helpful, they really fall over themselves to help you.’ (Female 3; worse)

‘… I joined a group in [town] that was erm, it’s called Sense and it’s for partially sighted and hearing people … You, you get out you mix, we make things.’ (Female 4; worse)

### Information needs

Despite having contact with a range of health professionals and allied health professionals, many of the participants did not feel they had adequate information about their condition and its prognosis. Several of them had been diagnosed and discharged from the eye clinic without further information.

‘And I was immediately discharged … … unless there was some change ‘cause they couldn’t do anything they said.’ (Female 6; better)

They acknowledged that, while the condition appeared to be fairly common, there was little access to information about it and often information was given with complex terminology, which only resulted in more confusion.

‘It sounds so common but yet nobody knows a lot about it.’ (Female 8; better)

With the information needs of the participants not being adequately met, they were largely relying on information from other people with the condition including friends and family.

‘Only went online to find out … well that what I’ve got there and it’s due to … if I can read it … What’s it say? Advance … oh … geographic atrophy is it? … Advanced dry eye, AMD in both eyes and it’s macular degeneration … mmm.’ (Male 1; better)

‘I was talking a friend the other day and I didn’t realise … and she said she’d got this she’s had it for 20 odd years and she said erm she said you don’t go blind she said, I haven’t lost my sight she said you’ve got peripheral vision, but it’s the central vision that goes. So you can ….’ (Female 7; better)

All the participants stressed the need for a support group where they could discuss their condition with others who were experiencing the same symptoms, as well as possibly ‘guest’ health professionals, who could provide information.

‘I mean, just talking to other people who are in the same boat erm.’ (Female 6; better)

‘But if you’ve got somebody here to say well … say look this is what happens and that’s why that’s been done and that’s why that’s been done I think you’d be on a winner.’ (Male 1; better)

‘I don’t think people you know, when you’re talking about it, understand exactly, what is happening, I don’t think they realise it’s as bad as what we think it is.’ (Female 2; worse)

## Discussion

This exploratory research sought to understand specifically the experiences of patients with GA and their families regarding the impact of the condition on daily living tasks including social activities, the impact on quality of life, and the types of informal and formal support they accessed. Key findings indicated that: participants had little knowledge of the mechanisms of GA but were aware of their prognosis; activities impacted by GA included management of daily activities and reading; emotions experienced included fear and frustration, and these frequently crossed over into their relationships with others; and access to formal support was mainly practical and information needs were largely unmet, with peer support being identified as important.

These findings resonate with some of the themes identified in the literature, in particular the ‘impact on activities’. The impact of GA on functional ability and independence is well-documented. Previous studies have highlighted the difficulties individuals have with shopping, food preparation and navigation (Moore 2000; Moore & Miller 2003; Stanford et al. 2009); however, it is not clear whether their included study participants were diagnosed with GA, wet AMD or both. The emotional impact of GA was described by our participants in terms of anger, frustration and fear. Such emotions have been highlighted in other qualitative studies of AMD (Cimarolli et al. 2012; Owsley et al. 2006). Again, clinical characteristics of the study population are not reported, but it is reasonable to assume that the emotional impacts described are experienced by individuals with both wet AMD and GA. The effect such emotions have on personal relationships was discussed by our participants. Frustration was exacerbated by an increasing lack of independence, and despite the good intentions of friends and family their ‘help’ with daily activities often resulted in further frustration. These findings were reflected in both the ‘better’ and ‘worse’ groups. Hassell et al. (2006) examined the impact of AMD on individuals through quality of life questionnaires (Impact of Vision Impairment and Short Form General Health Survey, SF-12). They concluded that even individuals with mild visual impairment report a significant impact on quality of life. Furthermore, they found no relationship between duration of vision loss and emotional reaction to vision loss.

A key finding from our study was the unmet information needs of the participants. Participants even reported the focus groups themselves to be valuable for providing peer support. Poor provision of support has been highlighted in a previous study examining patients’ experiences of information provision for AMD within the UK (Burton et al. 2013). More recently, Boxell et al. conducted a large cross-sectional survey of individuals with AMD, recruited via the Macular Society, to investigate whether the introduction of management guidelines by the Royal College of Ophthalmologists had brought improvements in healthcare experiences (Boxell et al. 2017). They concluded that at diagnostic consultations with eye-care professionals, or at GP consultations, information support and provision were low. The participants in our study often experienced confusion as to the reasons they had been referred to the eye clinic by, for example, their optician and GP. They appeared to be unsure as to whether their appointment was routine or emergency, but on attending were informed that nothing could be done to treat this condition. This raised false hope and resulted in frustration. Similar experiences have been reported by Burton et al. (2016). Our participants were unaware of the local support available to them, and this could have been a motivation for them attending the focus groups. Hassell et al. (2006) observed three quarters of their study population had not used low-vision rehabilitation despite moderate or severe vision loss, and no waiting lists to access such services. Whilst conclusions cannot be directly drawn between this and our study, it can be argued that receiving a diagnosis is difficult for individuals. Information is given to them about the condition, prognosis and support that are available to them locally. Our study echoes the findings of Boxell et al. (2017), and more work is required to ensure individuals are provided with information they can refer to at any point in the future as remembering and processing such information at the time of a diagnosis where no further treatment is offered can be difficult. Signposting to other charities, organisations and support networks will be of benefit to individuals and may result in reducing the psychosocial effects of AMD.

This study was not without limitations. Despite every effort to maximise participant numbers within the focus groups, interest in the study was low. Feedback from the supporting clinicians at the NHS Trust noted that potential participants found the prospect of travelling to the focus groups daunting and unmanageable. Due to the tight time schedule of the study there was only a six-week window to recruit potential participants. Supporting clinicians at the Trust strongly believe a longer recruitment period would have resulted in increased number of participants. It is possible that potential participants were reluctant to take part in a focus group but may have preferred a one-to-one interview. Future studies should consider different types of data-collection methods to encourage engagement from all eligible participants. Patient and public involvement (PPI) can help inform decisions regarding selection of data collection methods. It is not possible to determine whether data saturation was reached. Focus groups were held during the day, which may also have influenced recruitment.

There was a lack of ethnic diversity within the study population. It is unknown how some of the themes identified may differ based upon cultural variation. This is an under-researched area and warrants further exploration. Despite low numbers and a lack of ethnic diversity, this study provided a strong in-depth discussion amongst participants, producing themes which resonated with existing evidence. Participants were very willing to discuss their thoughts, emotions and experiences of living with GA. There was only one male participant within our study, and it is was not possible for us to explore whether there were any differences in experiences between genders.

There were some concerns within the study team about the potential distress of having heterogeneous focus groups (combination of ‘better’ and ‘worse’ participants). Heterogeneous groups would have allowed for exploration of differences in experiences as a direct result of differing visual abilities. This could have provided further insight into areas around adaptation and acceptance of GA. The ethical implications of this do need careful consideration, particularly surrounding advice and support following the focus group.

## Conclusion

This study has explored the impact of GA on individuals and their perceptions of the impact on family members. A key finding was the lack of awareness of information about the condition and access to support, such as low-vision rehabilitation services and charities. Future research could focus on family members and their perspectives on how GA has impacted upon them and their daily lives. It would also be interesting to identify the impact of GA on both formal and informal carers. In terms of formal support, signposting to agencies, such as charities and support groups is recommended. Our findings suggest that more should be done to direct individuals to such services. Participants expressed a desire to know more about their condition, and to be able to maintain a dialogue with others about their experiences and challenges. Whilst there may not be a clinical need to regularly monitor individuals with GA there did appear to be a ‘social’ need, with some individuals strongly advocating a role for peer support. Variable access to services was noted within our study, although it is not clear if this is reflective of the situation across the UK. This important qualitative work will help to inform future outcome measures for GA AMD interventions.

## Data Accessibility Statement

The datasets used and/or analysed during the current study are available from the corresponding author on reasonable request.

## Additional File

The additional file for this article can be found as follows:

10.22599/bioj.137.s1 Supplementary file. Focus group topic guide.

## Footnotes

A local charitable organisation for visually impaired people.
