# Supplementary material for: Patient Perspectives in Geographic Atrophy (GA): Exploratory Qualitative Research to Understand the Impact of GA for Patients and Their Families
Source: Br Ir Orthopt J. 2019 Nov 12;15(1):133–41. doi: 10.22599/bioj.137 (PMC7510394; doi:10.22599/bioj.137)
Supplement: Supplementary file. — Focus group topic guide. [file bioj-15-1-137-s1.pdf]

Focus group topic guide

PATIENT PERSPECTIVES IN GEOGRAPHIC ATROPHY (GA):

Draft Focus Group Schedule

Introduction

- Greet and thank everyone for coming.
- Introduce self (SB) and other facilitator (AH).
- Background:
    - Brief background to the study (This study is funded by Roche Products Limited and is being carried out in collaboration with University of Sheffield. The aim of the study is to explore and understand the impact of Geographic Atrophy in the UK for patients and their families. We will be carrying out two focus groups in Sheffield with patients from the Sheffield Eye Clinic)
- With your permission we would like to record the interview. This will help us to focus on the important things that you say. [SB/AH will also be making notes during our discussion.]
- Everything is completely confidential. Information shared in this group will not be passed on outside the group by participants or the research team.
- We will only use first names (or your preferred names) in our discussion, and there will be no way of identifying any individual when we write up the report of this research.
- Important that only one person talks at a time, and that you share everything you have to say with the group rather than just the person next to you.
- During the Focus Group we might discuss things that you may find difficult. You don't have to answer any questions that you find make you feel uncomfortable but at the same time, we're very interested in your experiences of the issues that we touch on today. If

there are any questions that you are uncomfortable with, or you find difficult to answer, we can move on so please don’t worry.

- The Focus Group will take us approximately one hour to complete.

Before we start ...

Provide a further copy of the information sheet to all participants and ask them to complete the consent form and demographic data slip.

Let’s start by:

[ice breaker question to get all participant voices on the recording]

Focus Group topic guide

1. What do you understand about your eye condition?

   [*prompts: what have you been told about your condition?]*

2. What services have you accessed about your condition?

   [*prompts: Who have seen in the eye clinic? Any referrals to the low vision clinic? Monitoring by optometrist/optician?*]

3. What is bothering you most about the condition at the moment?

4. What effect is your eye condition having on your life?

   [*prompts: ADL’s (tasks; driving; signing); Emotional effects (feelings changing over time; loss of confidence; anxiety; depression; independence) reading*]

5. What effect is your eye condition having on your social activities/networks?

6. What is the impact on your family members/informal carers?

[*prompts; taking to appointments; reduced social life; carer's health and wellbeing (work)*]

7. What worries/concerns or thoughts do you have, if any:-

   Currently … ?

   About your future? [*prompt: deterioration; co-morbidities; isolation*]

8. Who do you normally talk to about the effect of the condition on your life?

   Informal care: spouse; wider family; anyone else?

   HCPs: Specialist nurse; optician/optometrist; GP; anyone else?

   Charitable organisations or other support networks?

9. What information would you like to receive about your eye condition?

10. Where do you go to get information about your eye condition?

    [*prompts: Google, patient advice groups, retinal specialist, nurse, GP, other?]*

11. Is there anything else you would like to share about your experience or any important issues you feel were left out?

12. If you could change one thing about your eye condition, what would it be?

Close

Hand out leaflet / list of support networks and researcher details if they need further support or information.

Thank them for their time.
